# Supplementary material for: A scheduler for rhythmic gene expression
Source: Mol Syst Biol. 2025 Oct 10;21(12):8. doi: 10.1038/s44320-025-00155-9 (PMC12673066; doi:10.1038/s44320-025-00155-9)
Supplement: Supplementary file 1 — Appendix [file 44320_2025_155_MOESM1_ESM.pdf]

Appendix

A scheduler for rhythmic gene expression

Authors: Dimos Gaidatzis, Maike Graf-Landua, Stephen P. Methot, Michaela Wölk, Giovanna Brancati, Yannick P. Hauser, Milou Meeuse, Smita Nahar, Kathrin Braun, Marit van der Does, Sirisha Aluri, Hubertus Kohler, Sebastien Smallwood, Helge Großhans

Correspondence to: dimosthenis.gaidatzis@fmi.ch helge.grosshans@fmi.ch

Table of Contents:

Appendix Figure S1 p. 2

Appendix Figure S2 p. 3

Appendix Figure S3 p. 4

Appendix Figure S4 p. 5

Appendix Figure S5 p. 6

Appendix Figure S6 p. 7-8

Appendix Figure S7 p. 9-10

Appendix Table S1 p. 11

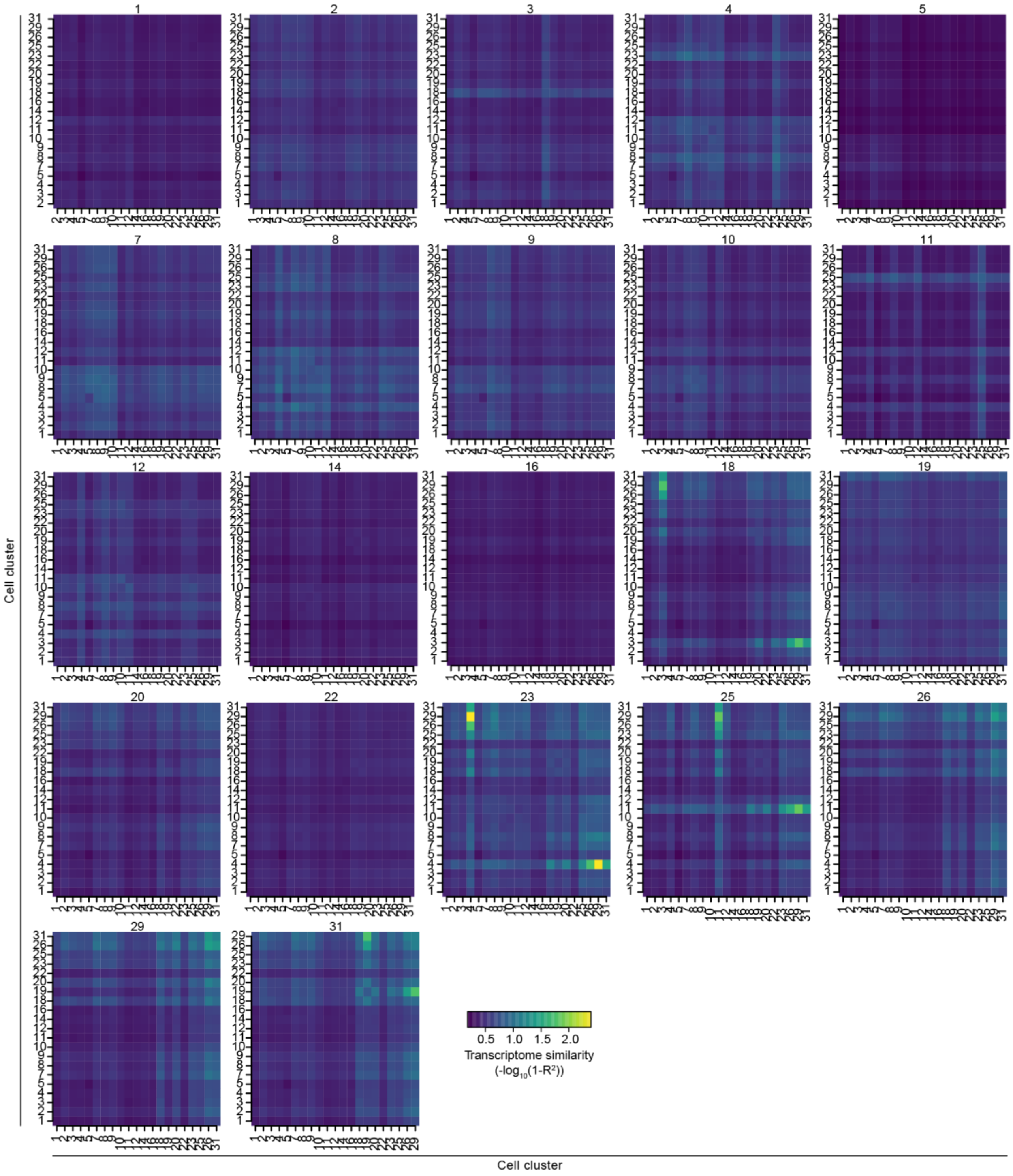

Appendix Figure S1: Detection of doublet clusters in scRNA-seq data.

To detect doublet cell clusters, each identified cluster (with >100 cells) was correlated against all possible two cluster combinations. Clusters 18, 23, 25 and 31 were identified as doublets and removed from subsequent analysis.

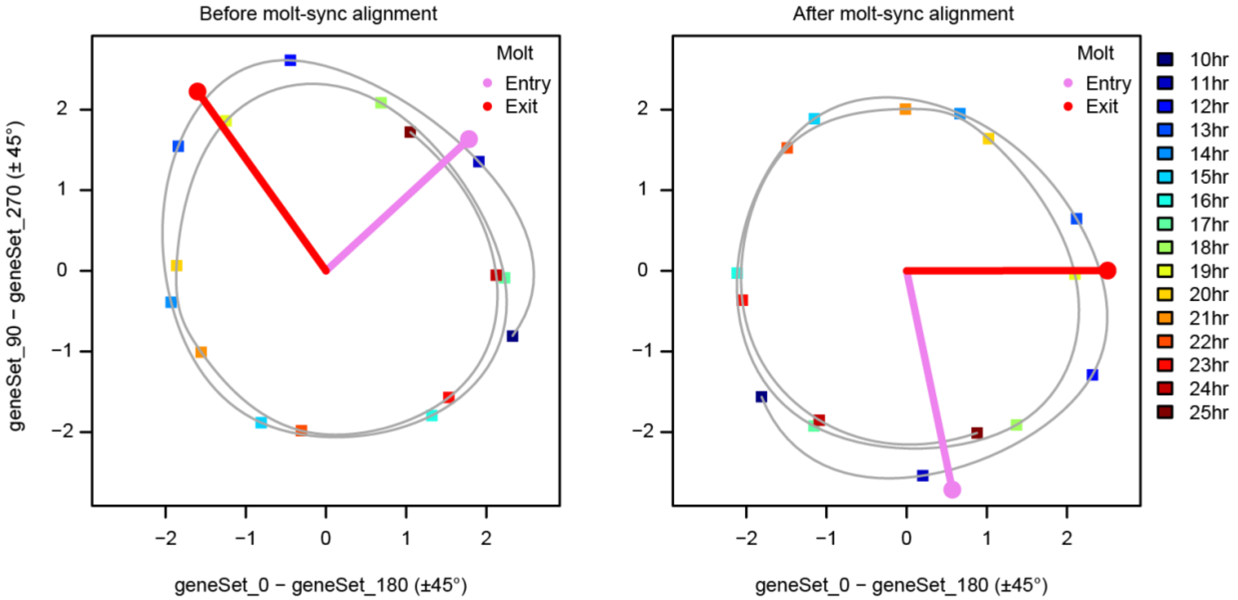

Appendix Figure S2: Molt-synchronization of time course RNA-seq datasets.

Scatter plots visualizing the developmental trajectory of a wild-type RNA-seq time course experiment (Meeuse et al., 2020) by plotting the average expression of orthogonal gene sets (Nahar et al., 2024) (see Methods). Briefly, this method uses prior peak phase information of 3,739 genes (Meeuse et al., 2020) to generate circular trajectories with time progressing in counter clock orientation (left panel). We used cubic spline interpolation performed on x and y separately to visualize the gray connecting lines between neighboring time points. Two additional RNA-seq samples were included in this analysis, namely a molt entry sample and a molt exit sample, where molt exit also defines the start of a new larval stage (see Methods). The position of the molt exit sample (125.8°) was used to rotate and thus calibrate the gene expression peak phases from (Meeuse et al., 2020) such that 0° represents the start of a larval stage. The right panel shows the same analysis as the left panel but using calibrated peak phase information (molt-sync).

A

Tissue	#Cells
Am/PH sheath cells	4
Excretory gland	2
Pharyngeal gland	5
Seam cells	32
Germline	N/A
Somatic gonad precursors	10
Vulval precursors	6
Distal tip cells	2
Unlabeled	N/A
Non-seam hypodermis	165
Excretory cells	3
Cholinergic neurons	N/A
Intestine	32
Coelomocytes	6
Intestinal/rectal muscle	4
Pharyngeal muscle	37
Body wall muscle 2	N/A
Body wall muscle 1	95
Pharyngeal epithelia	9

B

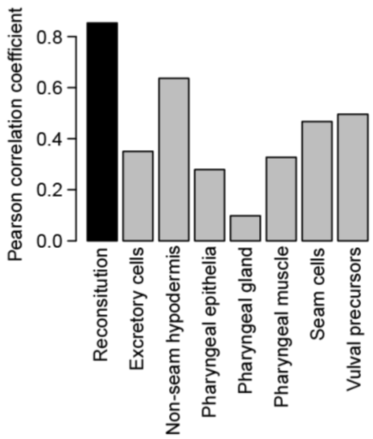

C

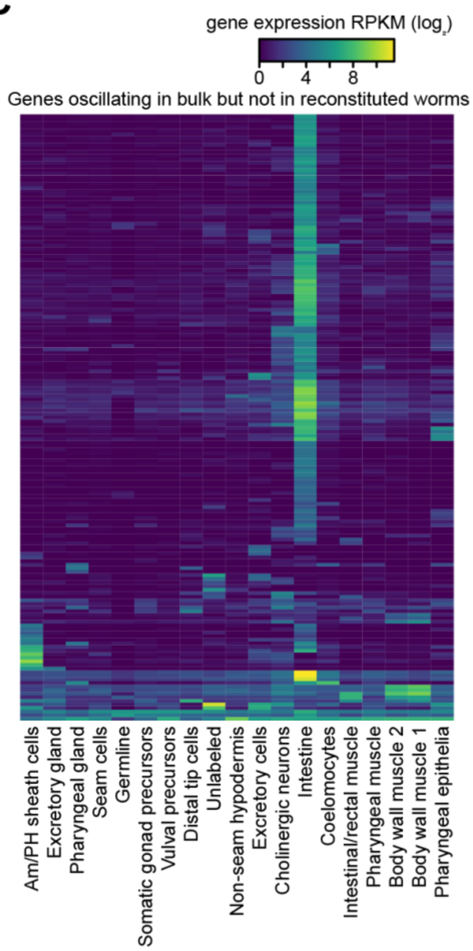

Appendix Figure S3: *In silico* reconstitution of bulk RNA-seq oscillations using scRNA-seq data.

A) Table indicating the number of cells per tissue in *C. elegans* larvae, based on (Altun and David, 2024), and used to determine the weight of each tissue in the bulk RNA-seq reconstitution. Specific tissues (labeled "N/A") were excluded from this analysis: Germline (nuclei are rapidly dividing), Unlabeled (not a defined tissue), Cholinergic neurons (low confidence due to general neuronal depletion), Body wall muscle 2 (redundant to body wall muscle 1).

B) Barplot of Pearson correlation coefficients from the comparison of gene expression amplitudes of the bulk RNA-seq dataset against either the *in silico* reconstituted dataset (see Fig.1F), or each oscillating tissue dataset alone.

C) Tissue-specific expression for a select set of genes that oscillate in the bulk dataset, but not the *in silico* reconstitution dataset (See Fig.1F - green dots). Heatmap shows the absolute $\log_2$ expression values for individual genes (rows) in individual tissues (columns), based on a *C. elegans* tissue atlas (Cao et al., 2017).

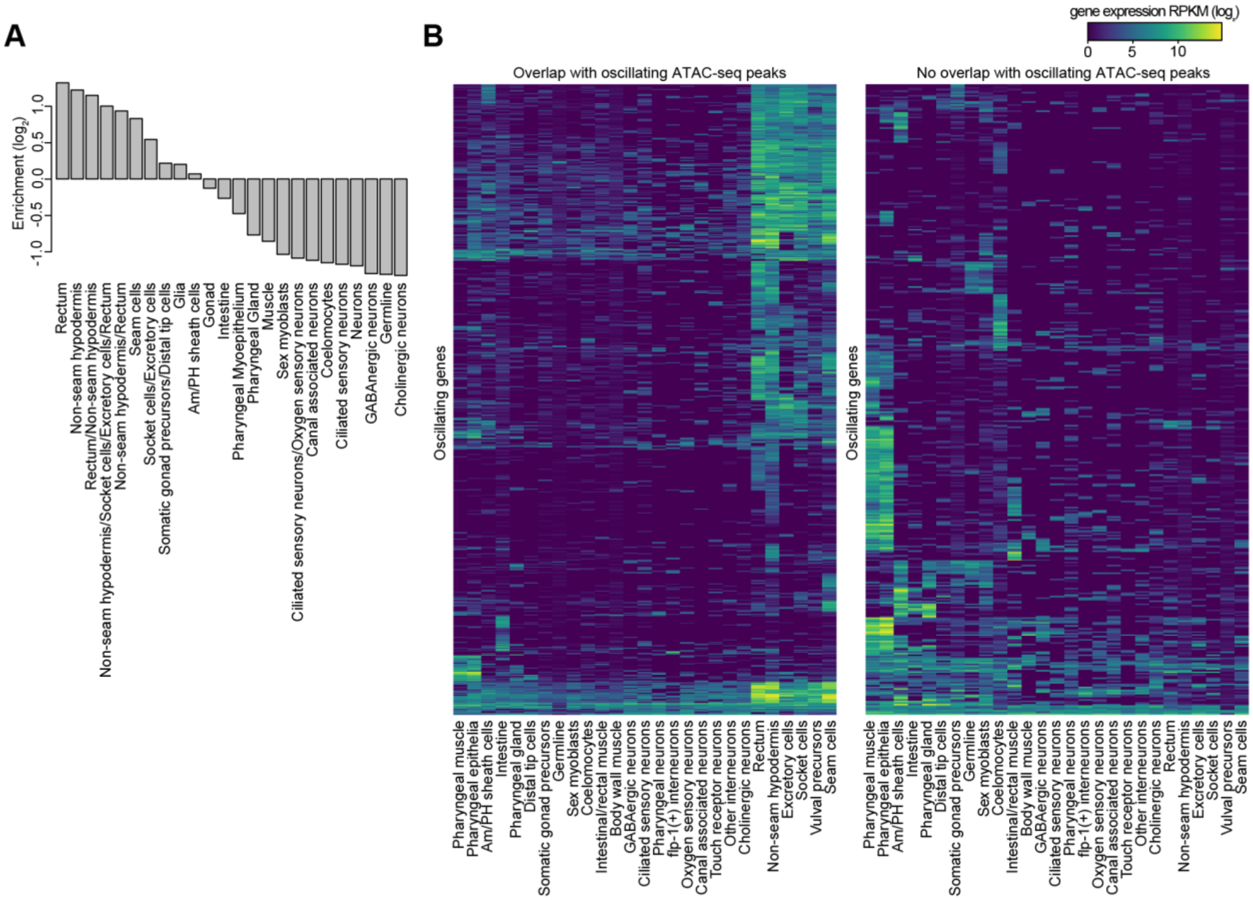

Appendix Figure S4: Tissue specificity of oscillating ATAC-seq peaks.

A) Barplot comparing the proportion of oscillating ATAC-seq peaks in each tissue. Overlapping our ATAC-seq data with scATAC-seq annotations from (Durham et al., 2021), we calculated the tissue-specific enrichment of oscillating peaks as $\log_2$ (oscillating peaks overlap / all peaks overlap).

B) Tissue specific expression of a select set of genes in the *C. elegans* tissue atlas (Cao et al., 2017). Left panel depicts genes that oscillate at the mRNA-level and at the ATAC-seq level (red dots – Fig.2C). The right panel depicts genes that oscillate at the mRNA level but are not associated directly with an oscillating ATAC-seq peak (bottom right dots – Fig.2C). Note the enrichment of pharynx-specific genes in the right panel, which is in line with our observation of incomplete pharynx dissociation during the nuclear isolation protocol (see Methods).

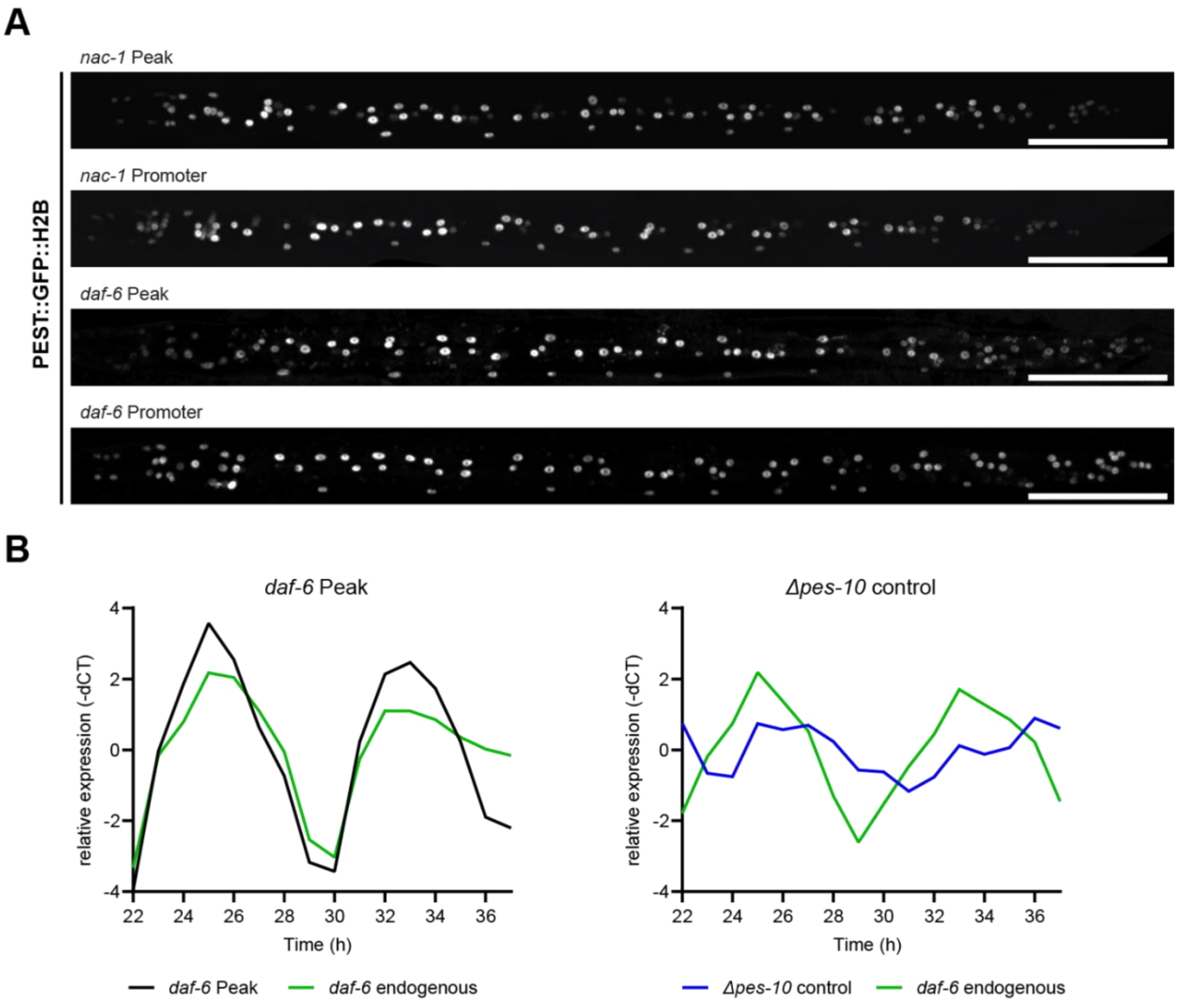

Appendix Figure S5: Tissue distribution and transcript dynamics of reporter constructs.

A) Representative maximum-intensity projection microscopy images of GFP expression from each reporter construct from Fig.3D,E. All animals are L4 stage and images selected around the peak of reporter expression. Brightness and contrast were adjusted independently for each image in order to compare the spatial expression pattern of each reporter construct. Scale bars, 100 µm.

B) RT-qPCR time courses, comparing expression of the endogenous *daf-6* transcript with *gfp* reporter transcripts driven by either the *daf-6* ATAC-seq peak upstream of the *Δpes-10* minimal promoter, or the *Δpes-10* minimal promoter alone. The endogenous *daf-6* transcript and *gfp* transcript were quantified from the same RNA preparations. Relative expression was calculated as –dCT = −(target CT values – actin CT values) and then mean normalized for each trace individually. 1 replicate was performed per reporter.

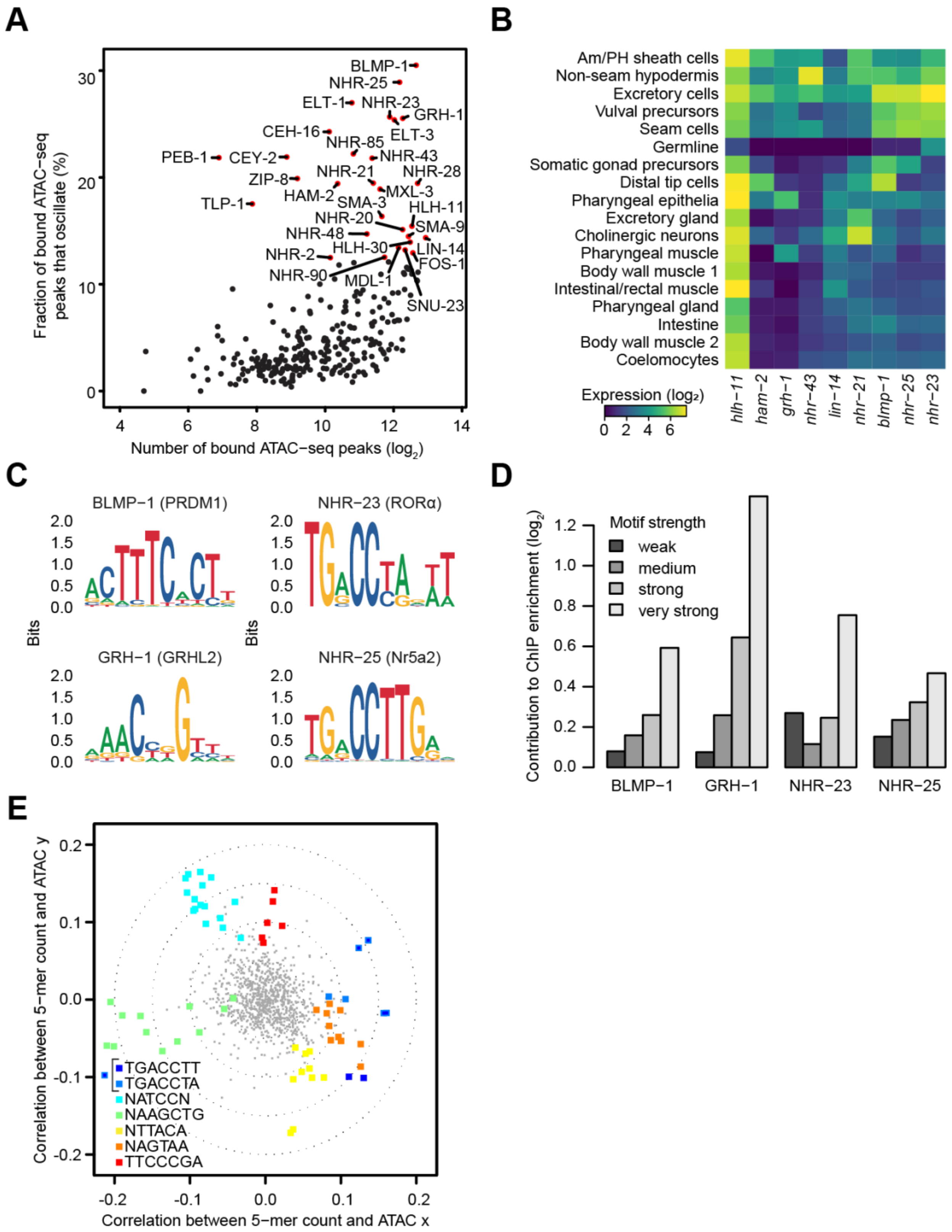
A
B
C
D
E
BLMP-1
NHR-25
ELT-1
NHR-23
GRH-1
ELT-3
CEH-16
NHR-85
PEB-1
CEY-2
NHR-43
ZIP-8
NHR-21
NHR-28
HAM-2
MXL-3
TLP-1
SMA-3
NHR-20
HLH-11
SMA-9
NHR-48
LIN-14
NHR-2
HLH-30
FOS-1
NHR-90
MDL-1
SNU-23
Fraction of bound ATAC−seq peaks that oscillate (%)
Number of bound ATAC−seq peaks (log2)
Am/PH sheath cells
Non-seam hypodermis
Excretory cells
Vulval precursors
Seam cells
Germline
Somatic gonad precursors
Distal tip cells
Pharyngeal epithelia
Excretory gland
Cholinergic neurons
Pharyngeal muscle
Body wall muscle 1
Intestinal/rectal muscle
Pharyngeal gland
Intestine
Body wall muscle 2
Coelomocytes
Expression (log2)
hlh-11
ham-2
grh-1
nhr-43
lin-14
nhr-21
blmp-1
nhr-25
nhr-23
BLMP−1 (PRDM1)
NHR−23 (RORα)
GRH−1 (GRHL2)
NHR−25 (Nr5a2)
Bits
Contribution to ChIP enrichment (log2)
Motif strength
weak
medium
strong
very strong
BLMP−1
GRH−1
NHR−23
NHR−25
Correlation between 5−mer count and ATAC y
Correlation between 5−mer count and ATAC x
TGACCTT
TGACCTA
NATCCN
NAAGCTG
NTTACA
NAGTAA
TTCCCGA

Appendix Figure S6: Enrichment of TF binding and motifs at oscillating ATAC-seq peaks.

A) Scatterplot intersecting ChIP-seq peaks from modERN (Kudron et al., 2018) and the ATAC-seq peaks. Each dot represents a TF. For each TF, the total number of ATAC-seq peaks overlapping with ChIP-seq peaks is indicated on the x-axis (log_2-transformed), while the percentage of overlapping ATAC-seq peaks that are oscillating is indicated on the y-axis. Labelled TFs (red dots) were above the background of 12.2% overlap with oscillating ATAC-seq peaks.

B) Heatmap of gene expression for the 9 molting clock TFs in all tissues from the scRNA-seq dataset.

C) Transcription factor motif logos derived using ChIP-seq data from mammalian homologs of molting clock TFs (from HOMER (Heinz et al., 2010)).

D) For each of the four TFs for which we have a high confidence motif (B), we devised a linear model to predict ChIP-seq enrichment at Osc+ ATAC-seq peaks using the number of weight matrix hits in the score bins of 7-8 (weak), 8-10 (medium), 10-12 (strong) and >12 (very strong). The y-axis represents the contribution of motif strength to ChIP-seq enrichment (coefficients).

E) Scatterplot showing for every possible 5-mer the Pearson correlation coefficient between the occurrence of a given 5-mer within Osc+ ATAC-seq peaks and the respective x- or y- coordinates (see Fig.4A). The correlation coefficients are mean centered on both axes. The color of a 5-mer indicates whether a particular 5-mer matches one of the sequences listed in the legend when considering both strands. The sequences in the legend were manually selected. The character N in the legend represents a position that can match any nucleotide. 5-mers that match multiple sequences in the legend are indicated.

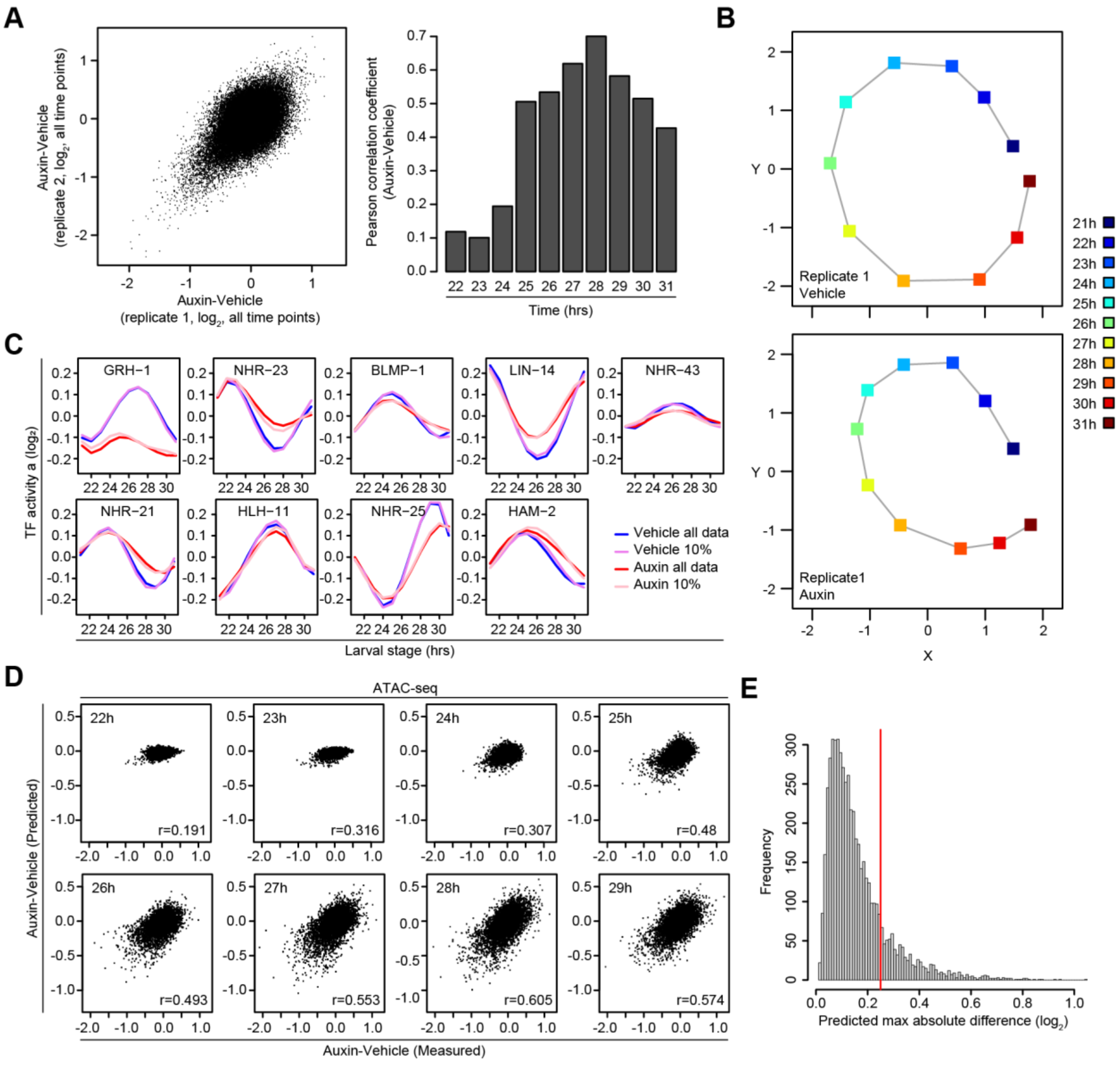

Appendix Figure S7: Predicting chromatin accessibility changes upon GRH-1 depletion.

A) *Left*, Scatterplot of differential accessibility in *grh-1::degron* animals (auxin - vehicle), compiling all peaks and all timepoints, comparing replicate 1 (x-axis) to replicate 2 (y-axis). *Right*, Barplot of Pearson correlation coefficients, quantifying the consistency of differential accessibility (Auxin-Vehicle) between the two replicate experiments at each timepoint separately.
B) Scatter plot visualizing oscillations in the RNA-seq time course datasets from *grh-1::degron* animals treated with vehicle or auxin (similar to Fig.S5C). The auxin treatment causes the trajectory to slow down before collapsing.
C) Linear model applied to the ATAC-seq time course at each timepoint independently (similar to Fig.6A). Model was fit using either the whole dataset

(vehicle blue, auxin red) or using only 10 % of the data (vehicle fuchsia, auxin pink) from the *grh-1::degron* transgenic animals. The model was performed independently for each timepoint such that TF activity represents the model coefficient for each TF at each timepoint. When the model is fit with only 10% of the data, the coefficients remain very similar.

D) Scatterplot of differential ATAC-seq signal (auxin - vehicle) at all ATAC-seq peaks used for modeling (Fig.4A), comparing predicted and measured changes at indicated timepoints after auxin/vehicle addition. Pearson correlation coefficient (r) is indicated at each timepoint.

E) Histogram showing the distribution of ATAC-seq peaks based on their largest (from all timepoints) predicted absolute difference in chromatin accessibility upon GRH-1 depletion. Red line at absolute difference = 0.25 indicates the threshold for selecting predicted differential peaks (see Fig.6C-E).

Appendix Table S1: Oligos used.

Target	Primer name	Primer sequence	Description	strain
cloning *daf-6* promoter	YPH133	GCGTGTCAATAATATCACTCTGTTAGTTGCAATCACCTC	Forward primer for Gibson Assembly of the *daf-6* promoter + pYPH0.14	HW3507
	YPH134	GCTAAGTCTAGACATAGAAAACCTGTAAAATACAGAAAC	Reverse primer for Gibson Assembly of the *daf-6* promoter + pYPH0.14	
cloning *daf-6* ATAC-seq peak	YPH221	GCTAAGTCTAGACATCTGAAAGTTAAAAATTACAG	Reverse primer for Gibson assembly of *daf-6* ATAC-seq peak + *Δpes-10* into pYPH0.14 (overhang with *Δpes-10* + pYPH0.14)	HW3508
	YPH222	GCGTGTCAATAATATCACTCCATATCTTCCCATCA	Forward primer for Gibson assembly of *daf-6* ATAC-seq peak + *Δpes-10* into pYPH0.14 (overhang with *daf-6* ATAC-seq peak + pYPH0.14)	
	YPH224	AATTTGCAAAAAATCGATAAACCAACTTGAACAAATATTG	Reverse primer for Gibson assembly of *daf-6* ATAC-seq peak + *Δpes-10* into pYPH0.14 (overhang with *daf-6* ATAC-seq peak + *Δpes-10*)	
	YPH225	TATTTGTTCAAGTTGGTTTATCGATTTTTTGCAAATTACG	Forward primer for Gibson assembly of *daf-6* ATAC-seq peak + *Δpes-10* into pYPH0.14 (overhang with *daf-6* ATAC-seq peak + *Δpes-10*)	
cloning *nac-1* promoter	MG130	gcgtgtcaataatatcactcTTTCGGCACCCTCAAAATCTC	Forward primer for Gibson Assembly of the *nac-1* promoter into plasmid pYPH0.14	HW3718
	MG131	CCATGGCTAAGTCTAGACATGATTGGAGGCTCTGCAATACTAG	Reverse primer for Gibson Assembly of the *nac-1* promoter into plasmid pYPH0.14	
cloning *nac-1* ATAC-seq peak	MG132	GCGTGTCAATAATATCACTCAAGGTACATCACAGATC	Forward primer for Gibson Assembly of the *nac-1* ATAC-seq peak into plasmid pYPH51	HW3719
	MG133	GTAATTTGCAAAAAATCGATGATTTATGGTGTAATACAC	Reverse primer for Gibson Assembly of the *nac-1* ATAC-seq peak into plasmid pYPH51	
qPCR for GFP	YPH120	ACAAGTGTTGGCCATGGA	Reverse primer for qPCR quantification of GFP	
	YPH121	CTTGTTGAATTAGATGGTGATGTT	Forward primer for qPCR quantification of GFP	
qPCR for endogenous *daf-6*	YPH170	CTATCACGAGGCCTTTCCA	Forward primer for qPCR quantification of endogenous *daf-6*	
	YPH171	CCCCACAACGTCATATAACCAAA	Reverse primer for qPCR quantification of endogenous *daf-6*	
