# Supplementary material for: A scheduler for rhythmic gene expression
Source: Mol Syst Biol. 2025 Oct 10;21(12):8. doi: 10.1038/s44320-025-00155-9 (PMC12673066; doi:10.1038/s44320-025-00155-9)
Supplement: Supplementary file 7 — Expanded View Figures [file 44320_2025_155_MOESM7_ESM.pdf]

Expanded View Figures

Figure EV1. Assigning and pseudo-timing oscillating tissues based on scRNA-seq.

(**A**) Correlation (R^2) of gene expression profiles from the cell clusters (rows) against the *C. elegans* tissues identified in (Cao et al, 2017). Clusters were assigned to the tissue with the highest correlation, except for cluster 10, which had a low correlation to multiple tissues and remained unassigned. (**B**) The expression of annotated oscillating genes (Meeuse et al, 2020) in all the identified tissues. Genes are ordered by 1D-tSNE. Left, scatterplot for the molt-synchronized phase of expression (degrees) of oscillating genes, indicated by the position of the dot along the *x* axis. Right, heatmap of expression levels for each oscillating gene (rows) in individual tissues (columns). (**C**) PCA plot comparing PC1 versus PC2 for seam cells, separating the cells into four panels according to their experimental timepoint. (**D**) Determining a tissue-specific and time-resolved expression profile for an example gene. A cubic smoothing spline (red circles) was fit for the gene *Y54G2A.76* on the scatterplot comparing the assigned pseudo-time, in PC space, versus normalized UMI counts per cell for each cell (black dots). Expression data shown is from the seam cell cluster. (**E**) Synchronization of the tissue and time-resolved expression profiles by comparison to an external bulk RNA-seq reference. For each tissue, we created variant expression profiles starting at different positions within the cycle and considering both clockwise (red) and counterclockwise (black) orientations. The panels display the Pearson correlation coefficients between the variant expression profiles and the bulk RNA-seq reference. (**F**) Correlation between experimental time and inferred pseudo-time. For each experimental timepoint, we determined the cell density along pseudo-time accounting for the calibration step illustrated in (**E**). The heatmaps show $\log_2$ cell density enrichments.

© The Author(s)

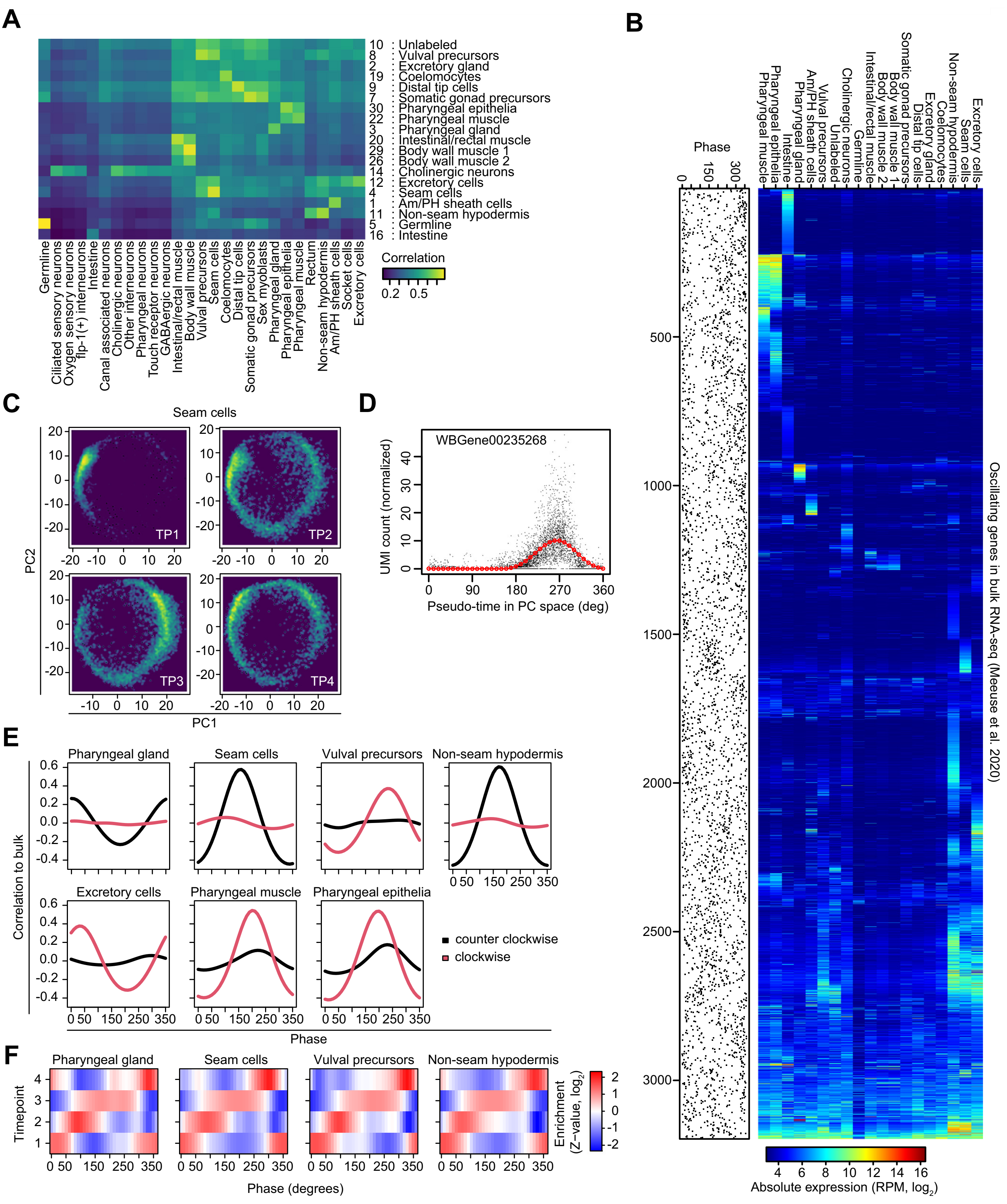
A
10 : Unlabeled
8 : Vulval precursors
2 : Excretory gland
19 : Coelomocytes
9 : Distal tip cells
7 : Somatic gonad precursors
30 : Pharyngeal epithelia
22 : Pharyngeal muscle
3 : Pharyngeal gland
20 : Intestinal/rectal muscle
29 : Body wall muscle 1
26 : Body wall muscle 2
14 : Cholinergic neurons
12 : Excretory cells
4 : Seam cells
1 : Am/PH sheath cells
11 : Non-seam hypodermis
5 : Germline
16 : Intestine
Correlation
B
Phase
Oscillating genes in bulk RNA-seq (Meeuse et al. 2020)
Absolute expression (RPM, log2)
C
Seam cells
TP1
TP2
TP3
TP4
PC1
PC2
D
WBGene00235268
UMI count (normalized)
Pseudo-time in PC space (deg)
E
Pharyngeal gland
Seam cells
Vulval precursors
Non-seam hypodermis
Excretory cells
Pharyngeal muscle
Pharyngeal epithelia
counter clockwise
clockwise
Correlation to bulk
Phase
F
Timepoint
Enrichment (Z-value, log2)
Phase (degrees)

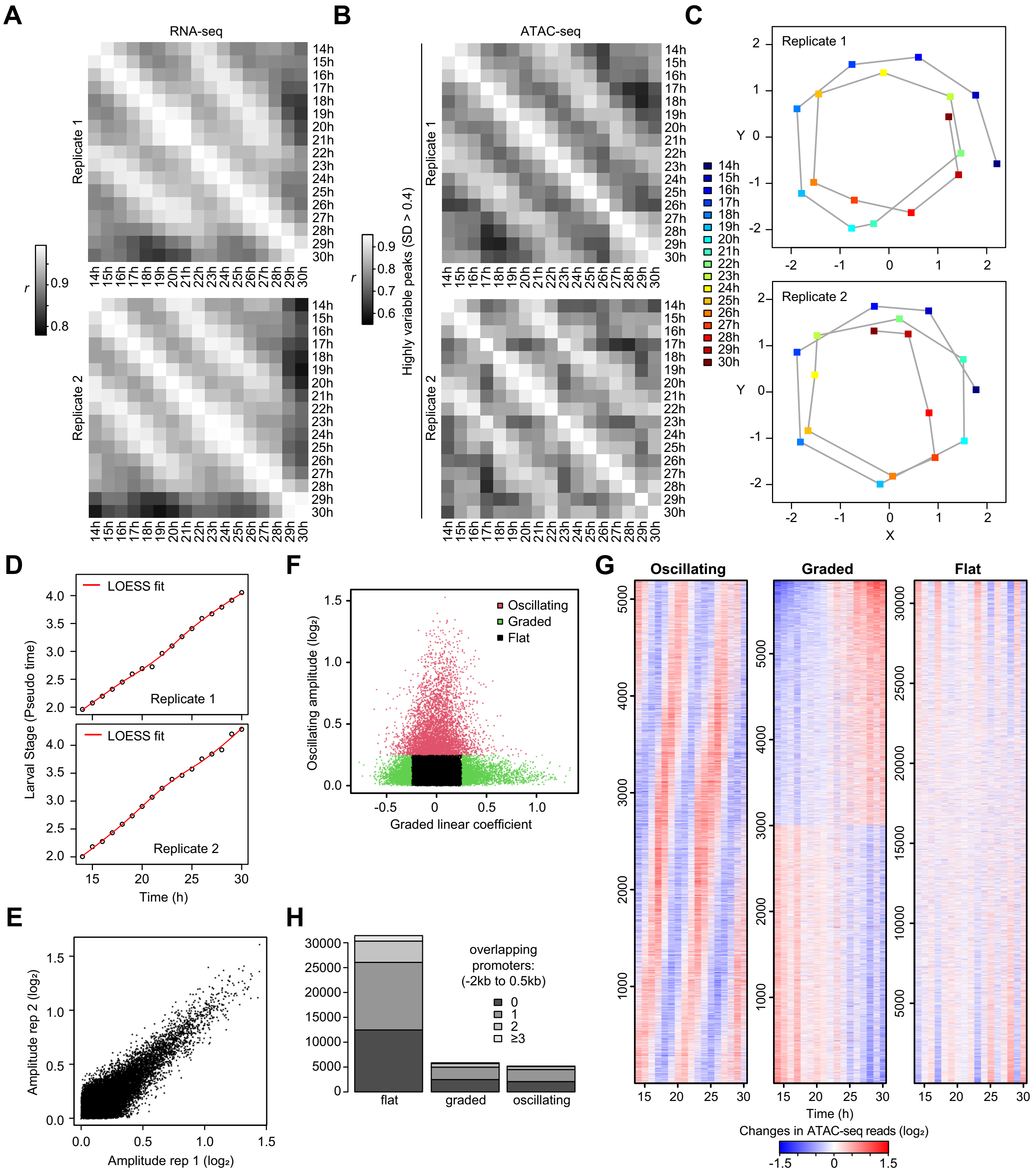
A
RNA-seq
Replicate 1
Replicate 2
r
B
ATAC-seq
Highly variable peaks (SD > 0.4)
C
Replicate 1
Replicate 2
X
Y
D
LOESS fit
Replicate 1
Replicate 2
Larval Stage (Pseudo time)
Time (h)
E
Amplitude rep 2 (log2)
Amplitude rep 1 (log2)
F
Oscillating
Graded
Flat
Oscillating amplitude (log2)
Graded linear coefficient
G
Oscillating
Graded
Flat
Time (h)
Changes in ATAC-seq reads (log2)
H
overlapping promoters: (-2kb to 0.5kb)
0
1
2
≥3
flat
graded
oscillating

 © The Author(s)

Figure EV2. Matched RNA-seq and ATAC-seq larval development time course.

(**A**, **B**) Pairwise correlation heatmap for log_2-transformed RNA-seq (**A**) or ATAC-seq (**B**) data in each of the 17 time points from the 2 independent replicates. Periodic changes in correlation indicate oscillations in both RNA-seq and ATAC-seq. r indicates the Pearson correlation coefficient. In (**B**), only highly variable peaks are used (SD > 0.4). (**C**) Scatter plot visualizing the developmental trajectories for the 2 replicate RNA-seq time course datasets. Trajectories were calculated similar to Appendix Fig. S2 (see "Methods"). (**D**) Scatter plot comparing the experimental time (*x* axis) to the unwrapped pseudo-time (*y* axis) for the 2 replicate RNA-seq time course datasets. We transformed the trajectories from panel (**C**) into polar coordinates to calculate the wrapped pseudo-time, which was unwrapped to obtain the final pseudo-time (*y* axis). A LOESS fit (red line) was used to smooth the pseudo-time. (**E**) Scatterplot comparing ATAC-seq amplitudes from replicate 1 versus replicate 2 for all peaks. Amplitudes were obtained by performing cosine fits on each replicate separately. (**F**) Cosine fitting of ATAC-seq peaks was used to identify their amplitude, which was plotted against the trend derived using a linear regression (trend is the graded linear coefficient). This reveals three distinct classes; graded (green), flat (black) and oscillating (red). (**G**) Heatmaps of mean-normalized and log_2-transformed changes in ATAC-seq reads at individual peaks over time. Peaks were classified according to their amplitude and graded linear coefficient (**F**). Oscillating peaks are sorted by their peak-phase, graded peaks by the graded component, and flat peaks by principal component 1. See Fig. 2A for replicate experiment. (**H**) Barplot of the total number of ATAC-seq peaks from each category that overlap the indicated number of promoters.

© The Author(s)

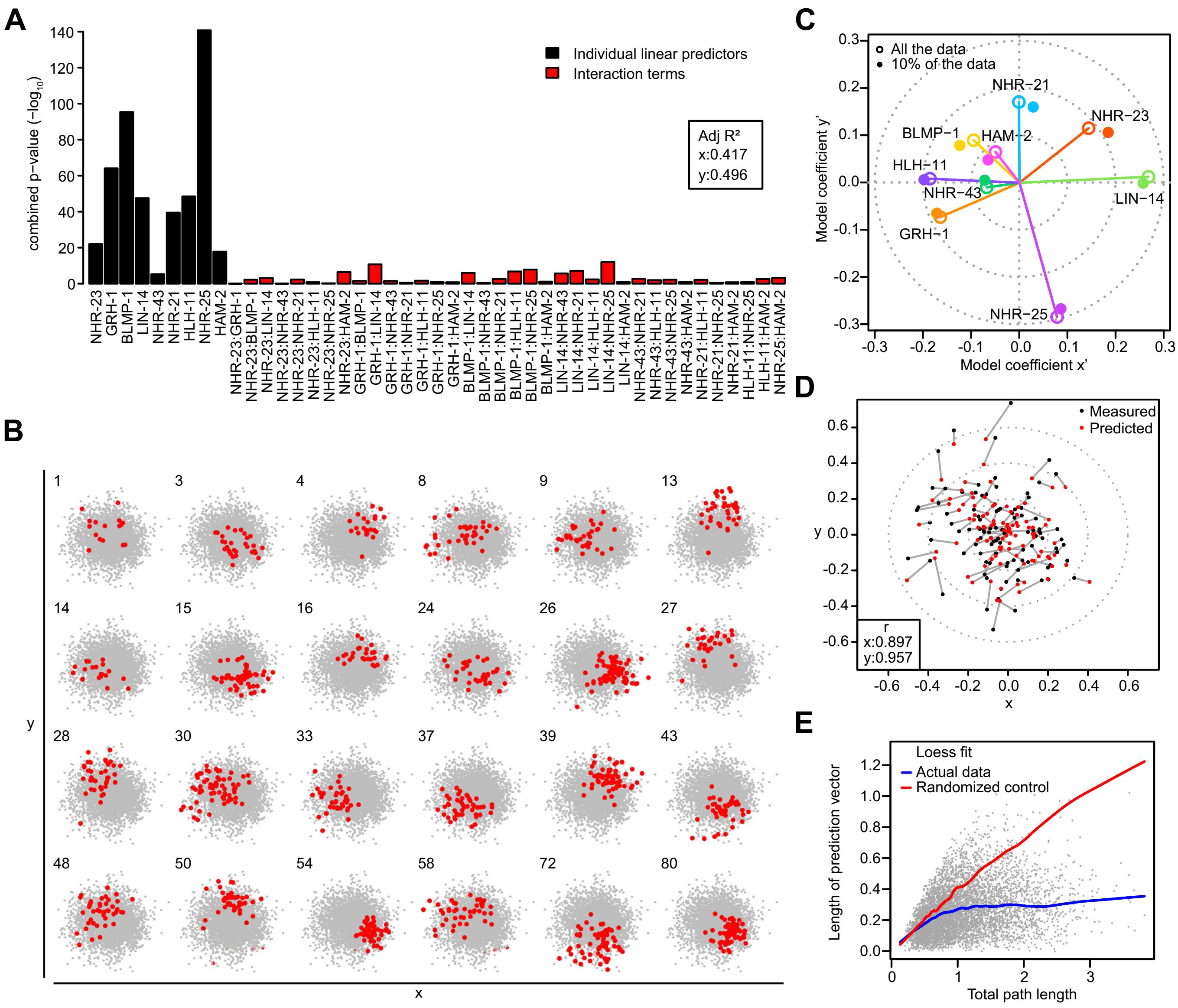

Figure EV3. Binding of 9 transcription factors is predictive of ATAC-seq phase and amplitude.

(**A**) Barplot indicating the relative influence of each molting clock TF on the linear model including pairwise interaction terms. Black bars indicate independent contributions, red bars contributions through interactions. The predictive power of the model (adjusted R^2) is indicated on the right. (**B**) Radar plots representing clusters depicted in Fig. 5C, generated based on ChIP-seq enrichments of molting clock TFs (cluster numbers are indicated in the top left corner). Each plot displays peak phase and amplitude (as x,y Cartesian coordinates) for all ATAC-seq peaks (gray dots), with cluster-specific peaks highlighted (red dots). (**C**) Representation of the output from a linear model (similar to Fig. 4E), generated using either 10% of ATAC-seq peaks (filled circles) or all peaks (empty circles). (**D**) Scatterplot comparing predicted and measured vectors for all 100 clusters from Fig. 5B. The Pearson correlation coefficient (r) for both x and y predictions respectively are indicated in the bottom left corner. (**E**) Scatterplot comparing the total path length (generated by summing up the absolute lengths of the individual phase-vectors for each TF) against the predicted vector length (generated after summing the phase-vector contributions of all TFs) for each ATAC-seq peak. This was done for all ATAC-seq peaks used in the modeling. A LOESS fit was applied to the data and is plotted (blue line). A control was generated by randomly combining the TF enrichments from all ATAC-seq peaks, and a second LOESS fit was applied (red line).

 © The Author(s)

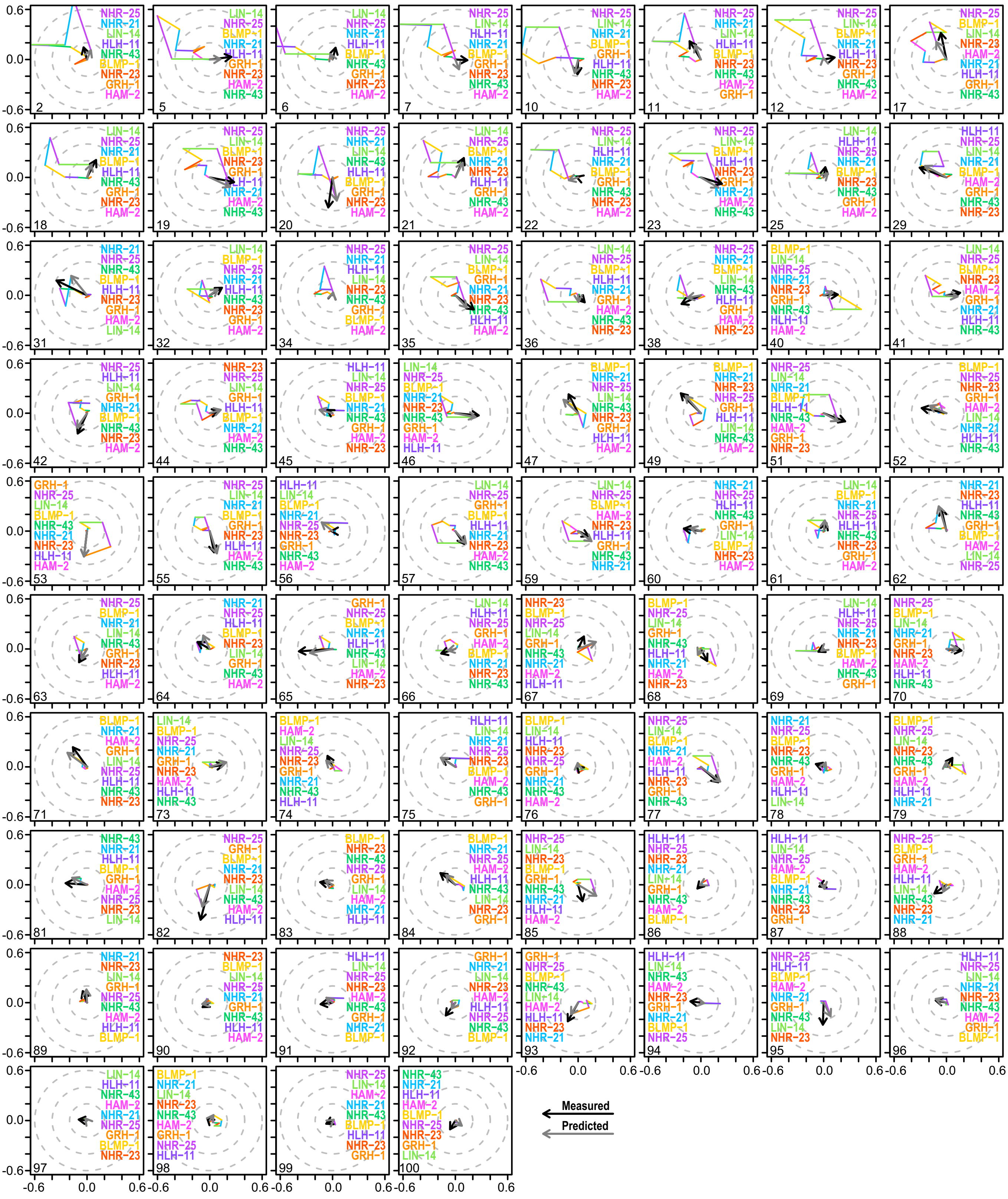
0.6
0.0
-0.6
2
5
6
7
10
11
12
17
18
19
20
21
22
23
25
29
31
32
34
35
36
38
40
41
42
44
45
46
47
49
51
52
53
55
56
57
59
60
61
62
63
64
65
66
67
68
69
70
71
73
74
75
76
77
78
79
81
82
83
84
85
86
87
88
89
90
91
92
93
94
95
96
97
98
99
100
Measured
Predicted

◀ **Figure EV4. Phase-vector predictions for remaining ATAC-seq clusters.**

Phase-vector predictions for individual ATAC-seq clusters (gray arrows) were generated by adding the phase-vector contributions (colored lines – average TF enrichment*x/y coefficients) of each TF. The average measured output vector for the cluster is indicated (black arrow). For each cluster, the phase-vectors are added together in ascending order, starting with the shortest phase, while the list of TFs is organized in descending order. Clusters are ordered based on their total path length (generated by adding the absolute length of each TF vector), aligned left to right, top to bottom. The 76 clusters not included in Fig. 5C are shown here.

 © The Author(s)

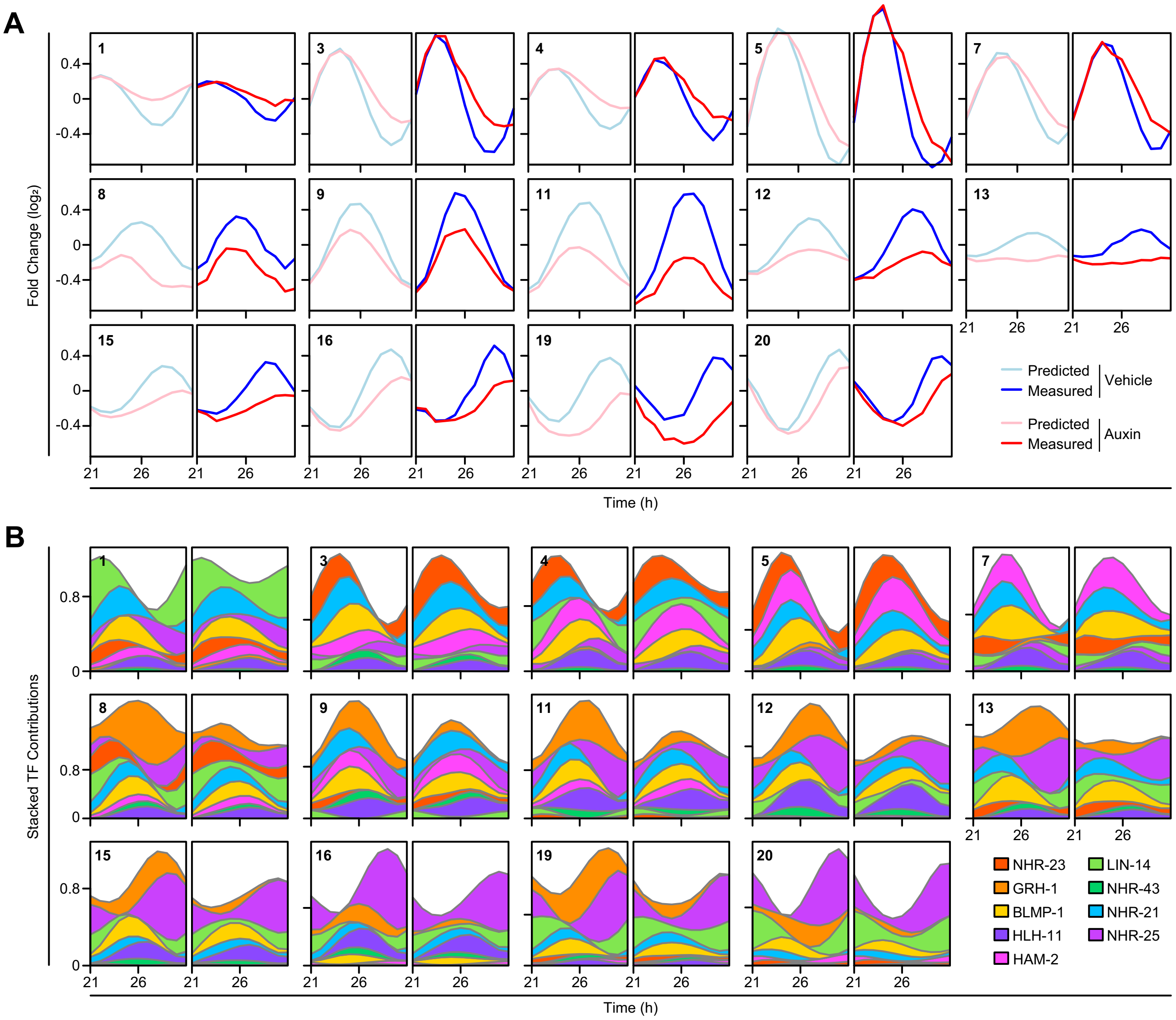

Figure EV5. Differential ATAC-seq predictions upon GRH-1 depletion.

(**A**, **B**) Depict clusters from Fig. 6C that were not highlighted in Fig. 6D,E and had more than 10 peaks. (**A**) Average predicted (left) or measured (right) accessibility changes over time for each cluster of differentially accessible peaks, comparing vehicle and auxin treatments. (**B**) Plots compiling individual TF contributions that generate the predicted chromatin accessibility over time. TFs are sorted by maximal activity difference between the vehicle and auxin conditions.

© The Author(s)
